# iMetaLab Suite: A one‐stop toolset for metaproteomics

**DOI:** 10.1002/imt2.25

**Published:** 2022-05-21

**Authors:** Leyuan Li, Zhibin Ning, Kai Cheng, Xu Zhang, Caitlin M. A. Simopoulos, Daniel Figeys

**Affiliations:** ^1^ School of Pharmaceutical Sciences, Faculty of Medicine University of Ottawa Ottawa Ontario Canada; ^2^ Ottawa Institute of Systems Biology University of Ottawa Ottawa Ontario Canada

**Keywords:** bioinformatics, database search, metaproteomics, microbiome, statistical analysis, visualization

## Abstract

Metaproteomics is a recently thriving technique that studies the collection of proteins in complex microbiomes of the human, animal, plant, and environment. The bioinformatics workflow required for metaproteomics research, from the database search and protein quantification to downstream functional and taxonomic analysis has been challenging and thus limiting the accessibility of metaproteomics to microbiome researchers. To overcome these challenges, we have developed a set of tools named iMetaLab Suite. iMetaLab Suite includes the following components: (1) MetaLab Desktop, an automated database search software that facilities proteins identification and quantitation from microbiomes; (2) the automated iMetaReport that allows users to quickly access database search results and data set profiles; and (3) an interactive online toolset, iMetaShiny, covering most frequently used functional, taxonomic, and statistical analysis in metaproteomics. iMetaLab Suite is a free, easily accessible, and actively updated toolset available to assist researchers to explore metaproteomic data.

## Highlights

- A one‐stop solution for metaproteomics data analysis for nonexpert.
- Database search and result reports that include taxonomy and function.
- Interactive tools for frequently used metaproteomics data analysis tools.

## Graphical abstract

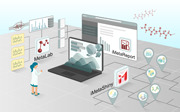

## INTRODUCTION

Proteins make up roughly 50% of the dry mass of microbial cells and play various roles in the microbes. Therefore, it is important to use proper techniques to understand the composition of proteins and the functional units of microbiomes. Metaproteomics is such a technique. Briefly, peptides derived from a protein extraction and digestion workflow are subjected to LC‐MS/MS analysis, and the resulting MS/MS spectra are compared with in silico generated theoretical spectra for peptide identification. This approach is easy to conduct for single‐species proteomics studies since the database is species‐specific and the size is ideal. For example, *Escherichia coli* strain K12 has a protein FASTA sequence database of 4375 protein sequences (1845 kB in size) from UniProt. However, when it comes to microbiome reference protein catalogs, database size increases dramatically to capture as many potential species as possible. As an example, the integrated gene catalog (IGC) database of the human gut microbiome has 9.9 million sequences and a size of 3.17 GB [1], that is, around 2000 times bigger than the *E. coli* strain K12 database. Using these large reference protein catalogs as databases, not only challenges computational capability but most importantly, negatively impacts the false‐discovery rate (FDR) modeling of the target‐decoy approach. To overcome this challenge, we previously developed the MetaPro‐IQ workflow that uses an iterative database search strategy to generate a reduced data set‐specific database for a MaxQuant search [2]. A conventional MaxQuant search output provides quantified peptide and protein group tables. Under the complex microbiome context, it is necessary, but challenging, to derive accurate taxonomic matches and comprehensive functional annotations from these search outputs. In addition, downstream data analysis and visualization of microbiome data adds an additional dimension of complexity compared to conventional proteomics, as both taxonomic and functional information are associated with the proteins. These challenges altogether make metaproteomics not easily accessible to scientists who are not experts in bioinformatics.

To overcome this challenge, we developed the iMetaLab Suite, which includes the entire framework of database search (MetaLab Desktop) for protein identification and quantification [3], an automated report (iMetaReport), and a variety of interactive tools for data analysis and visualization (iMetaShiny). iMetaLab was rooted from our previous MetaPro‐IQ workflow, the implementation of which required computational knowledges. Upon rising requests from scientists, we wrapped up the workflow into a desktop standalone version in which we eventually involved features of spectra clustering [3], posttranslational modification analysis [4], and built‐in iMetaReport modules. We share the toolset with the microbiome research community. iMetaLab Suite now has registered users from over 160 different institutions around the world. We aim to make iMetaLab Suite a free and one‐stop toolset for metaproteomics, with increasing amounts of tools under active development.

## RESULTS

### Overview of iMetaLab Suite

**Figure 1:**
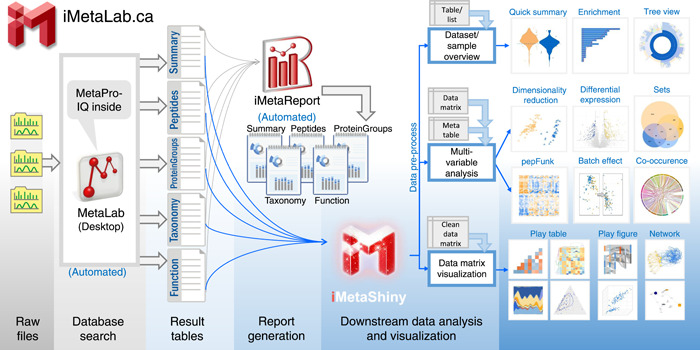
Framework of the iMetaLab Suite. Users load raw files to the MetaLab Desktop software to perform an automated metaproteomics database search. After the search, a series of result tables will be generated. Based on the search results, iMetaReport will be automatically generated, covering quick views of identification summary, peptides, proteinGroups, taxonomy, and function of the data set. Using the result tables, users can go to iMetaShiny for various types of downstream data analysis and visualization.

The iMetaLab Suite tools (Figure 1) are accessible through https://iMetaLab.ca. The MetaLab Desktop software can be freely downloaded from the website or through email requests sent to techteam.metalab@gmail.com to access the latest version. The software takes user input of LC‐MS/MS raw files, experimental design meta table (optional), workflow, and parameter settings. Detailed documentation of the MetaLab Desktop is accessible at https://wiki.imetalab.ca/. Under default settings, MetaLab will execute a database search and automatically generate result tables, including Summary, Peptide, ProteinGroup, Taxonomy, and Function tables that are frequently used in downstream analysis. Different formats of the taxonomy and functional results are generated to meet different data visualization requirements.

The iMetaReport is generated automatically following MetaLab database search. A pop‐up notice will be sent to the user to navigate to an html report. The iMetaReport contains five major tabs that statistically and visually summarize the Summary, Peptide, ProteinGroup, Taxonomy, and Function outputs, respectively. Optimal visualization is achieved when users input their experimental design (meta table) at the database search step. A sample iMetaReport can be accessed at: https://report.imetalab.ca.

The iMetaShiny apps are a collection of data analysis and visualization Shiny apps that are frequently applied in metaproteomics data analysis. The apps are divided into three subclasses based on their purposes. The first subclass of apps are for data set and sample overview, including Quick summary of LC‐MS/MS identification for quality checks, Enrichment analysis that includes both taxonomic and functional enrichment based on user‐input protein list or table, and Tree view based on user‐input NCBI taxonomic IDs. The second subclass of apps is for multivariate data analysis, including dimensionality reduction tools (PCA, PLS‐DA, and t‐SNE), differential protein expression analysis, Sets analysis, pepFunk [5], Batch effect explorer, and co‐occurrence analysis. The third subclass of apps is for data visualizations based on user‐input tables preformatted to meet the plotting requirements. For each of the Shiny apps, a sample data set is given to demonstrate the workflow and to guide users to prepare their input data table. We also provide a 96‐well plate randomizer and a Sample scrambler to aid users in their metaproteomics experimental design. More apps are being continuously developed and updated for access to the community.

### Case studies and results

#### Case I: Database search and automated report of data set overviews

**Figure 2:**
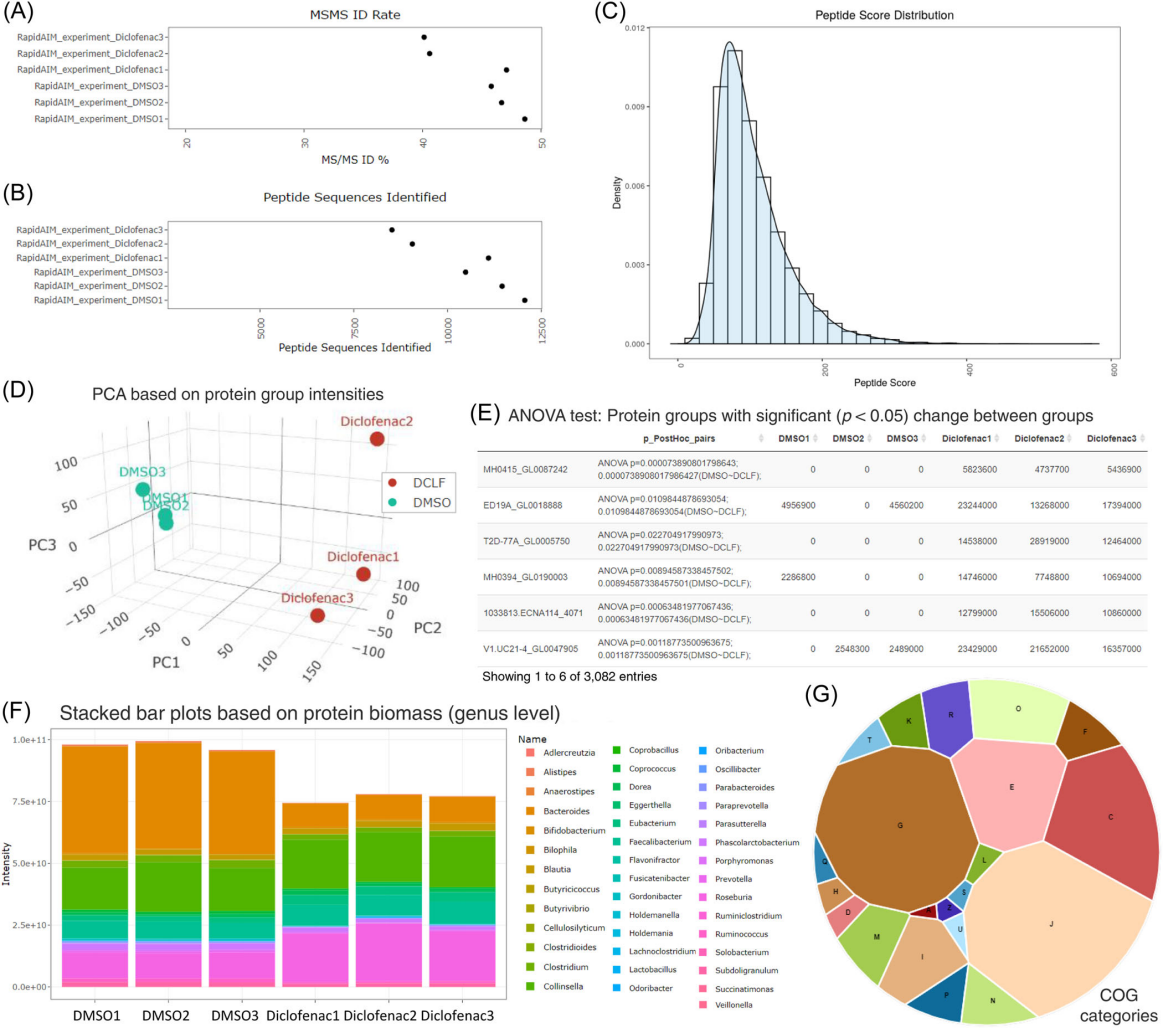
Examples from iMetaReport. (A) ID summary report: MS‐MS identification rate of each sample. (B) ID summary report: Number of peptide sequences identified in each sample. (C) Peptide report: Peptide score distribution in the data set. (D) ProteinGroup report: principal component analysis based on protein group intensities. (E) ProteinGroup report: analysis of variance test based on protein group intensities. (F) Taxonomy report: stacked bar plots based on protein biomass (genus level). (G) Function report: composition of clusters of orthologues categories in the data set.

One individual microbiome was cultured with or without the presence of diclofenac (an NSAID drug) in triplicates, the data set was taken from our previously published work [6]. The protein digests were analyzed using a 1.5‐h gradient with Orbitrap Q‐Exactive. MetaLab Desktop (V2.2) was used to search the six samples against the IGC database using the default settings of closed search. By using four threads on a Windows server (Two Intel Xeon E5649 processors, 96 GB RAM), this search took 14 h to complete. After the database search, a series of result files, including summary.txt, peptides.txt, proteinGroups.txt, and BuiltIn.taxa.all.csv, and functions.tsv, were generated. An iMetaReport was also automatically created. The report was presented as an html webpage consisting of five summary tabs for visualizing identification (ID), peptides, proteinGroups, taxonomy, and function. The ID summary results took the summary.txt as input. In Case I, results showed that there were 21,600 peptide sequences identified and 6601 protein groups quantified in total, with an average MS/MS identification rate of 44.9% (Figure 2A,B). Taking peptides.txt and proteinGroups.txt as inputs, respectively, both Peptide and ProteinGroup reports provided important parameters, such as peptide charge states, score distribution (Figure 2C), intensity distribution, and so on, for users to examine the overall quality of the data. Both reports also provided a heatmap and principal component analysis (PCA) score plots to visualize the experimental outcome. The visualizations are based on log_10_‐transformed peptide intensities and proteinGroup label‐free quantification (LFQ) intensities, respectively. For the proteinGroup PCA visualization, log_10_‐transformed LFQ‐intensities were imputed using a robust sequential algorithm to resolve possible data sparsity. In this example, the two groups showed a clear separation on PC1 (Figure 2D). In the ProteinGroup report, if users set up the meta‐information in the database search, analysis of variance (ANOVA) will be performed between the user‐input experimental groups based on LFQ‐intensities, and FDR‐adjusted *p* values are given for both matrix and pairwise comparisons (Figure 2E). In the Taxonomy report, the number of taxa identification, alpha and beta diversity, as well as stacked bar plots of microbial composition were provided. As an example, differences in genus‐level protein biomass contribution in response to diclofenac treatment can be clearly observed (Figure 2F). In the functional report, functional compositions at different levels using various functional databases, including clusters of orthologues (COGs), were visualized (Figure 2E), and heatmap and PCA visualizations were also provided. In case users did not set up the meta‐information during the database search, after the search, the user can remove the original report file, set up meta information, and click “run” again. MetaLab will check through all existing search files and skip the steps that have been performed, directly leading to a regeneration of the iMetaReport with updated meta information. A complete example of iMetaReport is available at https://report.imetalab.ca. Note that iMetaReport is aimed at quick sample overviews; it is recommended that the users perform further data analysis using iMetaShiny applications.

#### Case II: Differentially expressed protein groups and their taxonomy and functions

**Figure 3:**
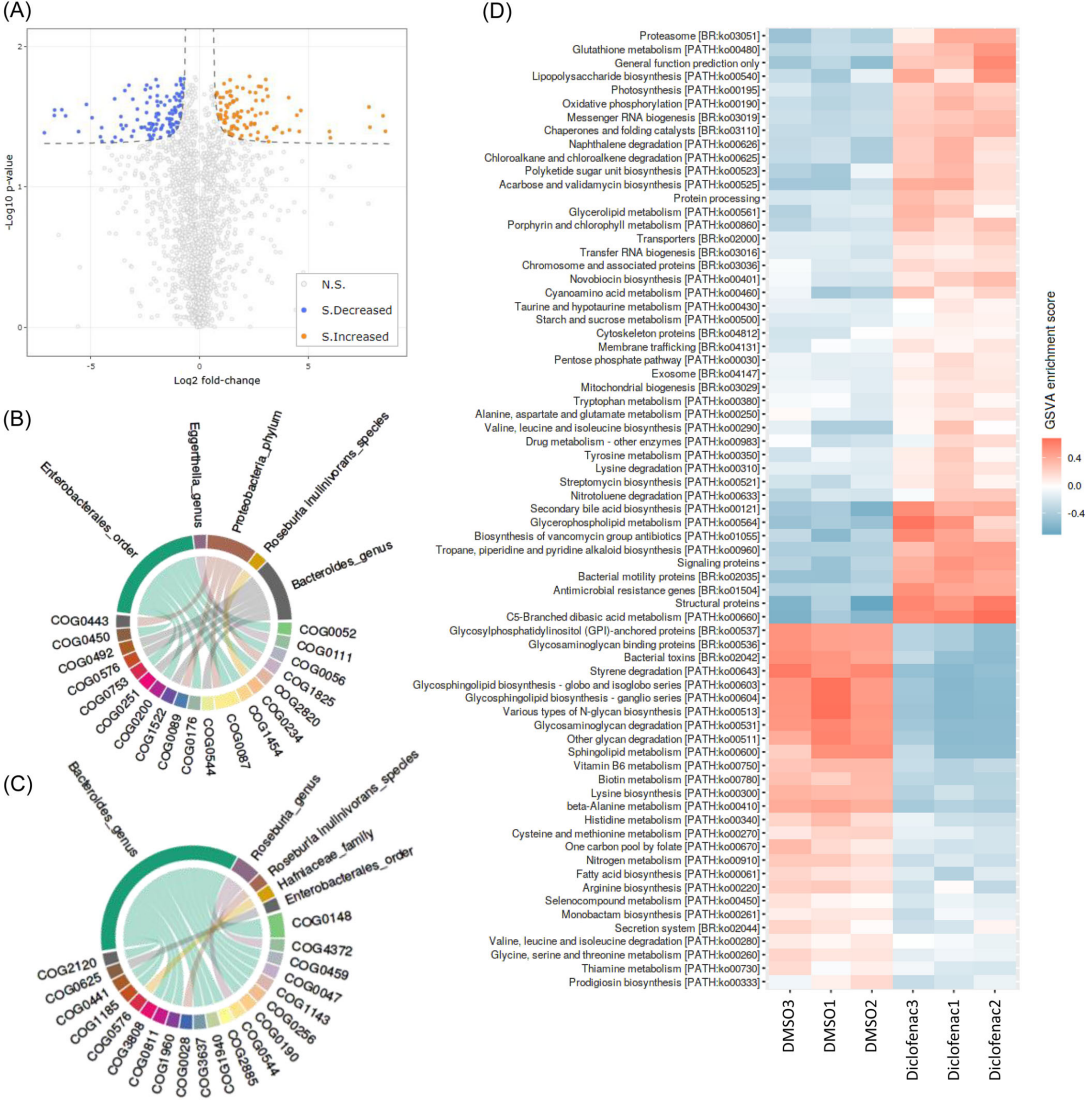
Examples of iMetaShiny applications. (A) Result of differential protein analysis from the diclofenac data set. Orange dots represent significantly increased protein groups, while blue dots represent significantly decreased protein groups. (B,C) Taxon‐function enrichment analysis of the significantly changed protein groups (using top‐1 protein in each protein group, *p* < 0.05). (D) Heatmap visualizing Gene Set Variation Analysis scores of the diclofenac data set.

Protein LFQ intensities from the search results of Case I were uploaded to the Differential Protein Analyzer (https://shiny.imetalab.ca/Volcano_plot/). The data preprocessing option was turned on and navigated us to the “Process Data” page. Here, we filtered out rows with 75% missing values and normalized them by columns. Users can also directly input their preprocessed protein expression table with the data preprocessing option kept off. We used default statistical parameters and a smooth‐curve threshold for determining the significantly changed protein groups. The resulting volcano plot is shown in Figure 3A. We obtained 95 significantly increased and 117 significantly decreased protein groups in response to diclofenac treatment in this metaproteomics data set. The table can be downloaded under the “Result table download” panel. Next, we examined the enrichment profile of the differentially expressed proteins. IDs of these proteins were uploaded to the Enrichment Analysis tool (https://shiny.imetalab.ca/metaproteomics_enrichment/), Function and Taxon correlation was selected as the analysis type, and COG was selected as the functional group type. Protein IDs were assigned with taxonomic and functional information, and we were navigated to the visualization page. Here, we chose to visualize the data using the Circos plot. As shown in Figure 3B,C, significantly increased protein groups are mainly from Enterobacterales, and genus *Bacteroides* had the most significantly decreased COG functions.

#### Case III: Peptide‐centric functional enrichment analysis

Besides using the LFQ protein group intensities, we demonstrate the peptide‐centric workflow through our pepFunk [5] (https://shiny.imetalab.ca/pepFunk/). The peptides.txt table was uploaded to the application, DMSO was set as the control, and diclofenac was set as the treatment. Using Gene Set Variation Analysis adapted for peptide data, significantly enriched KEGG pathways showed clear differentiation between the treatment and the control (Figure 3D).

## DISCUSSIONS

With iMetaLab Suite, we aim to maximize the accessibility of metaproteomic bioinformatics workflow to scientists with all levels of bioinformatics expertise in the field of microbiome research, as well as those in conventional proteomics/systems biology. We are actively developing novel database search workflows and strategies, as well as more statistical approaches for downstream functional, taxonomic, and ecological analysis of the metaproteomics data. These will be actively updated into the iMetaLab Suite and we welcome feedback and suggestions from users to improve the user experience and performance of the tools.

## METHODS

MetaLab Desktop is developed in Java and integrates open‐source third‐party libraries/tools MzJava [7], PRIDE Cluster [8], X!Tandem [9], MaxQuant [10], and Msconvert. iMetaReport is developed using R Markdown [11] with packages, including ggplot2 [12], plotly [13], tidyverse [14], vegan [15], ggdendro, d3heatmap, pheatmap, and so on. The server is hosted via openCPU [16] and therefore can be accessed publicly. User database search result is submitted by the MetaLab software to the openCPU server to generate the report. iMetaShiny apps are developed using R and the Shiny package [13], other frequently used packages are DT, data.table, shinyBS, htmlwidgets, and so on. It is hosted via shiny server. All these servers are hosted on Amazon cloud AWS.

## AUTHOR CONTRIBUTIONS

Daniel Figeys and Zhibin Ning conceptualized the framework of the iMetaLab Suite. Zhibin Ning established the webserver and bioinformatics frameworks for iMetaLab.ca, iMetaReport, and iMetaShiny. Zhibin Ning and Leyuan Li developed iMetaReports and iMetaShiny tools. Leyuan Li wrote the manuscript. Kai Cheng developed and maintains the MetaLab Desktop software. Xu Zhang developed the MetaPro‐IQ pipeline. Caitlin M. A. Simopoulos developed the pepFunk tool in iMetaShiny. All authors have tested the toolsets, revised the manuscript, read the final manuscript, and approved it for publication.

## CONFLICTS OF INTEREST

Daniel Figeys cofounded MedBiome, a clinical microbiomics company. Other authors declare no conflict of interest.

## Data Availability

All LC‐MS/MS sequencing data have been deposited to the ProteomeXchange Consortium via the PRIDE partner repository under submission number PXD033624. The database search results and reports are saved in GitHub (https://github.com/northomics/iMetaLab_paper). Supporting Information materials (graphical abstract, slides, videos, Chinese translated version, and update materials) may be found in the online DOI or iMeta Science http://www.imeta.science/.
